# Thoracic Aortic Aneurysm Surgery in Marfan Patients: a Perspective from the UK

**DOI:** 10.21470/1678-9741-2019-0214

**Published:** 2020

**Authors:** Amer Harky, Matthew Shaw, Mohamad Bashir

**Affiliations:** 1 Department of Cardiothoracic Surgery, St Bartholomew’s Hospital, London, UK.; 2 Department of Cardiothoracic Surgery, Liverpool Heart & Chest Hospital, Liverpool, UK; 3 Department of Research & Innovation, Liverpool Heart & Chest Hospital, Thomas Drive, Liverpool, UK.

**Keywords:** Marfan Syndrome, Aortic Valve Insufficuency, Aneurysm, Dissecting, Hemorrhage, Treatment Outcome

## Abstract

**Objective:**

Cardiovascular complications in Marfan patients include progressive aortic root dilation which can precipitate acute aortic dissection, ruptured aorta, severe aortic regurgitation, or all the aforementioned. Such complications can be fatal and the cause of death prior to any surgical intervention. We set out to identify the Marfan population in England and Wales and present their surgical outcomes.

**Methods:**

A total of 306 patients with Marfan syndrome who underwent aortic root surgery were identified between April 2007 and March 2013 from NICOR database. We examined the perioperative characteristics of such cohort along with in-hospital outcomes and survival.

**Results:**

Root and ascending segment procedures on Marfan patients performed in 3.3% of the total cohort by NICOR root surgery patients. The median reported age was 40 years (IQR = 29-49 years) and 100 (32.7%) were female. Of the patients analysed, 17.3% were treated non-electively and 68.6% of them received concomitant valve procedure. The in-hospital mortality was 2.0%. Reoperation for bleeding was required in 8.2% of patients and 1.3% of them suffered a cerebrovascular accident (CVA). Mortality at 1 year was reported as 5.5%.

**Conclusion:**

The outcomes of surgery on the root and ascending aorta in Marfan patients in the United Kingdom are satisfactory; however, the overall complexities of this patient population are not well understood and would benefit from further investigations.

**Table:** 

Abbreviations, acronyms & symbols	
AVOOMP	= Aortic valve operative outcomes in Marfan patients
BMI	= Body mass index
CCS	= Canadian Cardiovascular Society
COPD	= Chronic obstructive pulmonary disease
CVA	= Cerebrovascular accident
IQR	= Inter-quartile range
MFS	= Marfan syndrome
NACSA	= National Adult Cardiac Surgery Audit
NHS	= National Health Service
NICOR	= National Institute for Cardiovascular Outcomes Research
NYHA	= New York Heart Association
ONS	= Office for National Statistics

## INTRODUCTION

Marfan syndrome (MFS) is an autosomal dominant connective tissue disorder with key features that primarily affects the cardiovascular system, the eyes and the skeleton^[1]^. The reported birth incidence is estimated to be 1 in 9800 births^[2]^. It is well established that 50 to 80% of such patients will develop aortic dilation at some stage of their life^[3]^. Progressive aortic dilatation is often associated with aortic valve incompetence, which can lead to acute aortic dissection or even ruptured aorta, which can be life-threatening. Due to such predictable progression, MFS has been the base for extrapolation of clinical findings and daily practice to the thoracic aortic aneurysm that could be of different aetiologies^[4,5]^.

However, despite advanced understanding of the aetiology and pathophysiology of MFS, there is a lack of concrete approach in the current surgical management with 100% satisfactory outcomes. Indeed, except for modifications in surgical approaches and imaging assessments, all the treatment protocols that are in place in the current era were initiated between 1960 and 1970^[6]^. On the contrary, the surgical recourse and post-operative screening of susceptible patients meant that we now offer patients with MFS broad options to help promote patients’ quality of life and ensure long-term survival. It is also important to mention that medical therapeutics became a well-established avenue to halt progression of the disease. Furthermore, it was previously perceived that MFS patients would not be suitable candidates for endovascular approaches to an aortic aneurysm^[7]^. This exception has been challenged, and surgical endovascular therapy allowed itself a pace in this connective tissue disease. In this study, we wanted to reflect upon the experience of open surgical intervention on MFS patients in England and Wales, concentrating on procedures involving the root and ascending segments of the aorta, and to present the outcomes and results which will allow us to assess the current trend and direction of surgical correction of thoracic aortic aneurysm disease in this entity in the United Kingdom.

## METHODS

### NICOR Database Set

Data from the National Institute for Cardiovascular Outcomes Research (NICOR) and National Adult Cardiac Surgery Audit (NACSA) registry related to cardiac surgery in UK were extracted in November 2014. Records that were duplicate and in the form of percutaneous transcatheter aortic valve implantations were removed. Relevant data were cross-checked with each local unit for validation.

The inclusion criteria for this study were: procedures performed on the aortic root or ascending aortic parts with Marfan syndrome aetiology, performed in England or Wales between 1^st^ April 2007 and 31^st^ March 2013.

### Baseline and Operative Data

The data collected on each patient included patient pre-operative characteristics, medical conditions, operative data and post-operative outcomes. Pre-operative data included patient age, calculated at time of the procedure, patient gender, body mass index (BMI), angina classification according to the Canadian Cardiovascular Society (CCS), dyspnoea grade (as defined per the New York Heart Association - NYHA - grade <III and NYHA grade ≥III), history of recent myocardial infarction within 90 days of the actual surgical intervention, previous cardiac surgery, diabetes mellitus, current smoking status, presence of pulmonary disease or chronic obstructive pulmonary disease (COPD), hypertension, serum creatinine level of >200 µmol/L at time of surgery, any previous evidence of severe neurological dysfunction, presence of extracardiac arteriopathy, pre-operative heart rhythm, current left ventricular ejection fraction (classified as <30 poor, 30-50 moderate and >50% good), use of IV inotropes pre-operatively, presence of preoperative cardiogenic shock, and the operative urgency status. Operative data included the performance of concomitant revascularization and/or valve procedures, duration of aortic cross-clamp and cardiopulmonary bypass times.

### Outcome Variables

The primary outcome for this study was the in-hospital mortality rate. This is defined as any death that is due to any cause (cardiac or non-cardiac) during the same admission for cardiac surgery. The secondary outcome was in the form of mortality rate at time of at the 3-year period follow-up.

The in-hospital data were collected by the NACSA clinical registry system, while post-discharge survival rate was collected through linking patient records through their National Health Service (NHS) numbers directly to the Office for National Statistics (ONS) death registry; this office bears all deaths in England and Wales. The last follow-up date reported was 30^th^ July 2013.

Data on the causes of death were not available; however, an attempt to find these data through the in-hospital mortality data was made through linking patient records directly to the ONS registry prior to applying the extraction criteria.

Additional morbidity data according to NACSA definitions is also included in this analysis; these data were collected and validated in the same way as the pre-operative and operative data.

### Statistical Analysis

**Fig. 1:**
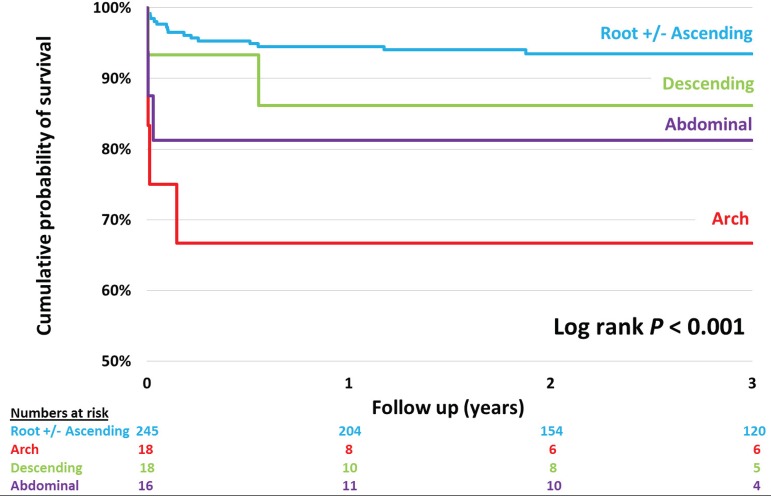
Kaplan-Meier chart showing 3-year survival stratified by the most treated distal aortic segments.

**Fig. 2:**
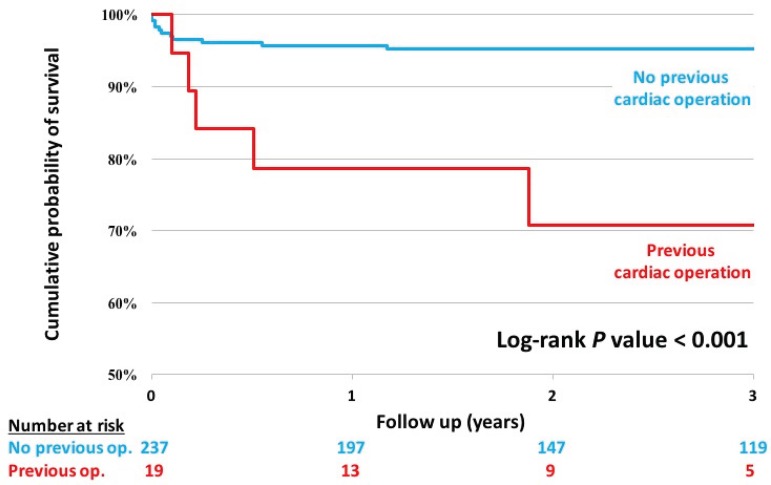
Kaplan-Meier chart showing 3-year survival in aortic root +/- ascending aorta surgery, stratified by history of cardiac surgery.

Data are summarized in the form of categorical and dichotomous variables; Figures 1 and 2 are summarised as event absolute number and percentage rate. If the continuous data were not normally distributed, then they are summarised as median and inter-quartile range (IQR). The number of missing data was very low; therefore, the categorical variables were recorded together with the baseline category list, while the continuous variable data were analysed together with the mean value prior to the statistical analysis.

Kaplan-Meier charts were used to actuarial 3-year survival (Figures 1 and 2). The log-rank test was used to assess the equivalence of mortality rates between groups.

Statistical analyses were performed using SAS version 9.3 (SAS Institute, Cary, NC). In all cases, a *P* value <0.05 was reported as statistically significant.

## RESULTS

### Characteristics of the Study Population

A total of 9144 patients were extracted from the NACSA database who underwent surgery on an aortic segment during the reported period of 2007-2013. Of these patients, 306 (3.3%) were recorded as having an aetiology of MFS and having a procedure on either the aortic root or the ascending aorta. However, 50 (16.3%) patients lacked the proper follow-up data in terms of mortality and were excluded from this analysis.

The cohort of 306 patients was treated at 38 different cardiac surgery, with a total of 106 different cardiac surgeons. The largest number of procedures performed by a surgeon during this period reported to be 15 patients; however, 50 surgeons (47.2%) operated on a single MFS patient. The largest number of procedures performed in a single hospital was 24. Tables 1 and 2 show the pre-operative and operative characteristics of this cohort.

**Table 1:** Pre-operative characteristics.

	Marfan syndrome(n=306)	Missing data
Age at operation (years)	40 (29, 49)	0 (0)
Female gender	100 (32.7)	0 (0)
Body mass index (kg/m2)	24.2 (21.1, 27.8)	5 (1.6)
Angina class IV	9 (2.9)	1 (0.3)
NYHA class ≥III	49 (16.0)	1 (0.3)
Previous Q-wave MI	6 (2.0)	0 (0)
Recent MI (within 90 days)	4 (1.3)	0 (0)
Previous PCI	2 (0.7)	7 (2.3)
Previous cardiac surgery	25 (8.2)	13 (4.3)
Diabetes (diet or insulin controlled)	5 (1.6)	0 (0)
Current smoker	38 (12.4)	7 (2.3)
History of hypertension	116 (37.6)	4 (1.3)
Creatinine >200 µmol/L	0 (0)	19 (6.2)
History of renal dysfunction	0 (0)	2 (0.7)
History of pulmonary disease	19 (6.2)	0 (0)
History of neurological disease	10 (3.3)	3 (1.0)
History of neurological dysfunction	6 (2.0)	2 (0.7)
Peripheral vascular disease	50 (16.3)	0 (0)
Non-sinus heart rhythm	14 (4.6)	10 (3.3)
Triple vessel disease	2 (0.7)	54 (17.7)
Left ventricular ejection fraction 30-50%	34 (11.1)	10 (3.3)
Left ventricular ejection fraction <30%	7 (2.3)	10 (3.3)
Intravenous nitrates or any heparin	10 (3.3)	0 (0)
Intravenous inotropes prior to anaesthesia	3 (1.0)	2 (0.7)
Pre-operative ventilation	4 (1.3)	3 (1.0)
Pre-operative cardiogenic shock	7 (2.3)	2 (0.7)

Continuous variables shown as median (25^th^ percentile, 75^th^ percentile); categorical variables shown as frequency (%). MI=myocardial infarction; NYHA=New York Heart Association; PCI=percutaneous coronary intervention.

**Table 2:** Operative details.

	Marfan syndrome(n=306)	Missing data
Elective operation	253 (82.7)	0 (0)
Urgent operation	21 (6.9)	0 (0)
Emergency operation	30 (9.8)	0 (0)
Salvage operation	2 (0.7)	0 (0)
****Procedure type****		
Composite valve graft and coronary reimplantation (modified Bentall or Cabroll)	147 (48.0)	0 (0)
Preservation of native valve and coronary reimplantation	95 (31.0)	0 (0)
Interposition tube graft with/without extension into the arch	36 (11.8)	0 (0)
Tube graft + separate AVR	14 (4.6)	0 (0)
Homograft root replacement	5 (1.6)	0 (0)
Aortic patch graft	4 (1.3)	0 (0)
Reduction aortoplasty	3 (1.0)	0 (0)
Sinus of Valsalva repair	2 (0.7)	0 (0)
Concomitant CABG procedure	20 (6.5)	13 (4.3)
Concomitant valve procedure	210 (68.6)	6 (2.0)
Concomitant 'other' cardiac procedure	70 (22.9)	12 (3.9)
Cardiopulmonary bypass time (mins)	166 (128, 214)	5 (1.6)
Aortic cross-clamp time (mins)	126 (97, 161)	6 (2.0)

AVR=aortic valve replacement; CABG=coronary artery bypass grafting

The median age reported was 40 years (IQR = 29-49 years), and 100 (32.7%) were female. In the cohort, 17.3% of the patients were treated on a non-elective base and 61.1% of patients had a concomitant valve procedure.

Twenty-five (8.2%) patients had undergone cardiac surgery during a previous hospital admission. Both previous and current surgeries for these patients are shown in Table 3.

**Table 3:** History of previous cardiac operations

	Previous cardiac operation (n=25)
****Previous procedure type****	
Valve + ascending aorta or aortic arch surgery	12 (48.0)
Isolated valve	6 (24.0)
Isolated ascending aorta or aortic arch surgery	3 (12.0)
Valve + 'other' cardiac procedure	2 (8.0)
Valve + ascending aorta or aortic arch surgery + 'other' cardiac procedure	1 (4.0)
'Other' cardiac procedure	1 (4.0)
****Current procedure type****	
Composite valve graft and coronary reimplantation (modified Bentall or Cabroll)	10 (40.0)
Interposition tube graft with/without extension into the arch	7 (28.0)
Aortic patch graft	3 (12.0)
Preservation of native valve and coronary reimplantation	2 (8.0)
Homograft root replacement	1 (4.0)
Tube graft + separate AVR	1 (4.0)
Reduction aortoplasty	1 (4.0)

AVR=aortic valve replacement

### Outcomes

The all-cause in-hospital mortality rate for all MFS patients undergoing aortic root or ascending aorta surgery was 2.0% (6 patients). No patients suffered paraplegia, 4 (1.3%) patients had post-operative CVA and 3 (1.0%) patients had transient ischemic attack (TIA). Twenty-five patients (8.2%) had reoperation for bleeding or tamponade; 1-year follow-up mortality was 5.5%. Table 4 summarises these outcomes.

**Table 4:** Post-operative outcomes.

	Marfan syndrome(n=306)	Missing data
In-hospital mortality	6 (2.0)	0 (0)
All cause reoperation	29 (9.5)	16 (5.2)
Reoperation for bleeding/tamponade	25 (8.2)	16 (5.2)
Neurological complications		
Paraplegia	0 (0)	29 (9.5)
CVA	4 (1.3)	29 (9.5)
TIA	3 (1.0)	29 (9.5)
Post-operative dialysis	4 (1.3)	22 (7.2)
1-year mortality	14/256 (5.5)	50 (16.3)

CVA=cerebrovascular accident; TIA=transient ischemic attack

Figure 2 shows a survival comparison stratified by patient history of previous cardiac operations, demonstrating a significant survival benefit for patients undergoing a first-time procedure (*P*<0.001).

## DISCUSSION

Elective aortic operations in MFS has improved life expectancy, regardless of the form or mode of surgery offered. The advent of composite root replacement, albeit mechanical or biological conduit, have promoted the armamentarium we now dialect to patients. Despite the cloud surmounting valve-sparing interventions and the risk of reoperation for aortic insufficiency, currently reported at 1.3% per year^[8]^, centres across the globe have reported improved results following valve-sparing root replacements in the MFS population^[9]^.

Surgical interventions are moving at a much rapid pace than the much-debated issues hauling timing of surgery, size, diameter, valve morphology and post-operative screening in MFS. The Aortic Valve Operative Outcomes in Marfan Patients (AVOOMP)^[10]^study group provided data on 316 patients that undergone aortic root replacement between 2005 and 2010. Total root replacement was performed in 24% (n=77), circulatory arrest was used in 29%; the median cardiopulmonary bypass time was 152 minutes, while the median cross-clamp time was 115 minutes. Valve-sparing interventions were performed in 76% of patients (n=239), circulatory arrest was used in 20%; the median cardiopulmonary bypass time was 194 minutes, while the median cross-clamp time was 156 minutes. Patients that underwent root operations were few in number, older in age and higher risk patients, which reflects the choice for valve-sparing surgery among these surgeons. The reoperation rate among the valve-sparing cohort was much higher. Putting all those issues aside, it is imperative, regardless of the form of surgery, that an operation should be offered to those patient populations to avert the detrimental aortic dissection.

In the UK, we drew conclusions from type A aortic dissection on national scale related to surgeon and hospital volume^[11]^. We provided evidence that a mean annual volume of less than four cases of type A performed by aortic surgeon is ill-fated. Faced with connective tissue disorder patients as in MFS and in centres that lack structured aortic referral, resources and concentration of aortic expertise, the much-feared MFS population will also have an ill fate not only in an emergency dissection setting, but also in the elective phase.

This study revealed that the overall in-hospital mortality rate in MFS patients who had aortic root surgery is 2.0%. When we zoom in, we find that only a small group of patients, *i.e*. 31% of the whole cohort, had native valve preservation. This surely raises a striking question: why is native valve-sparing surgery not being performed at much-extended breadth? We have no meaningful answer that we could divulge. However, as in the AVOOMP study, 2.5% of the cohort had problems during valve-sparing procedure, which resulted in conversion to total aortic root replacement with a composite graft. However, this outcome is not always reported in patients with valve-sparing interventions. This might explain the paradox we mentioned above as to perhaps surgeons in the UK are attempting the native valve preservation procedure explicitly and, due to lack of expertise, conversion to composite valve conduit ensue the high proportion of composite root repair seen in the results section. In a brief overview, conversion rate in the literature from intended valve-sparing to total aortic root replacement is at a higher rate than the AVOOMP rate of 1:40^[8]^, especially in small series cohort with less experienced surgeons.

The recent revision of the clinical guidelines likely will lead to a reduction in the rate of acute aortic root dissections in MFS patients. Nevertheless, aortic dissection can occur in the absence of any significant or known risk factors. Therefore, improvement in long-term prognosis of MFS patients can be achieved more accurately through earlier detection of progressed disease by the application of the current clinical guidelines, thorough assessment and follow-up imaging of the entire aorta, and looking into other risk factors that can predispose to aortic dissection. However, in the UK there is a lack of centralization of expertise and aortic service provision in all of its forms. Additionally, surgery on MFS patients is not being practiced through a rigorous protocol, which can affect the outcomes between high and low-volume surgeons^[12-14]^.

### Limitations

The retrospective nature of this study is the main limitation of this study. The main confounding factors are case selection, lack of data for structured referral and long-term follow-up through imaging modality to delineate the effect of natural history on the aorta. Other confounding factors include delays between diagnosis and initiation of treatment, lack of prophylactic operation data and referral bias. Furthermore, the direct effect of beta-blockers on the growth rate of the aortic root aneurysms has not been analysed and, as our cohort were prior to establishing such treatment, the results could have been different with the inclusion of this analysis.

## CONCLUSION

The nationwide reported results on aortic aneurysm surgery in MFS still represent a challenge. The aortic root +/- ascending aorta outcomes are close to the reported series in the literature. We call for an extended multidisciplinary team approach to deal with connective tissue disorders related to aortic disease and for such cohort to be concentrated through a robust referral system to aortic centres that provides a multitude of resources, and surgical expertise for aortic surgery in connective tissue disorders.

**Table:** 

Authors' roles & responsibilities	
AH	Substantial contributions to the conception or design of the work; or the acquisition, analysis, or interpretation of data for the work; drafting the work or revising it critically for important intellectual content; agreement to be accountable for all aspects of the work in ensuring that questions related to the accuracy or integrity of any part of the work are appropriately investigated and resolved; final approval of the version to be published
MS	Substantial contributions to the conception or design of the work; or the acquisition, analysis, or interpretation of data for the work; drafting the work or revising it critically for important intellectual content; agreement to be accountable for all aspects of the work in ensuring that questions related to the accuracy or integrity of any part of the work are appropriately investigated and resolved; final approval of the version to be published
MB	Substantial contributions to the conception or design of the work; or the acquisition, analysis, or interpretation of data for the work; drafting the work or revising it critically for important intellectual content; agreement to be accountable for all aspects of the work in ensuring that questions related to the accuracy or integrity of any part of the work are appropriately investigated and resolved; final approval of the version to be published
